# More focal, less heterogeneous? Multi-level meta-analysis of cathodal high-definition transcranial direct current stimulation effects on language and cognition

**DOI:** 10.1007/s00702-022-02507-3

**Published:** 2022-05-18

**Authors:** Jan Ostrowski, Jennifer Svaldi, Philipp A. Schroeder

**Affiliations:** 1 Department of Psychology, University of Tübingen, Tübingen, Germany; 2 Department of Systems Neuroscience, University Medical Center Hamburg Eppendorf, Hamburg, Germany

**Keywords:** High-definition tDCS, Cathodal tDCS, Transcranial direct current stimulation, 4 × 1, Cognition, Language, Anodal-excitatory cathodal-inhibitory framework

## Abstract

High-definition transcranial direct current stimulation (HD-tDCS) is a relatively focal, novel non-invasive brain stimulation method with the potential to investigate the causal contributions of specific cortical brain regions to language and cognition. Studies with HD-tDCS typically employ a 4 × 1 electrode design with a single central target electrode surrounded by four return electrodes, among which return current intensity is evenly distributed. With cathodal HD-tDCS, neural excitability in the target region is assumed to be reduced, which offers interesting perspectives for neuropsychological research and interventions. This multi-level meta-analysis compiles published studies using cathodal HD-tDCS in 4 × 1 configuration to modulate cognition and behavior. Regarding HD-tDCS, 77 effect sizes were gathered from 11 eligible reports. We extended this database with 52 effect sizes from 11 comparable reports using conventional tDCS with cathodal polarity. We observed no significant overall effect and no moderation by within-study and between-study variables in HD. In the extended analysis, results suggested a non-linear moderation of cathodal tDCS effects by intensity, driven by negative effect sizes at 1.5 mA. However, studies varied tremendously in task parameters, outcomes, and even technical parameters. Interestingly, within-study heterogeneity exceeded between-study heterogeneity in the present sample, and moderators hardly reduced the residual heterogeneity. Across domains and configurations, both positive and negative effect sizes are possible. We discuss the findings in relation to conventional cathodal tDCS and the framework of polarity specificity. Fundamental aspects of cathodal HD-tDCS are still to be addressed in future research.

**Supplementary Information:**

The online version contains supplementary material available at 10.1007/s00702-022-02507-3.

## Introduction

Since 1998, non-invasive electric stimulation of the brain with transcranial direct current stimulation (tDCS) has increasingly become a key methodology for causal inferences in neuroscientific research and gained immense interest over the past two decades (Nitsche and Paulus 2000; Polanía et al. 2018). Neuromodulation with tDCS has the potential to non-invasively alter diminished and excessive brain activity for fundamental research and interventions in patient groups (Lefaucheur et al. 2017; Polanía et al. 2018; Schroeder & Plewnia 2016). For instance, impaired working memory functioning in schizophrenia can be improved by anodal stimulation of the prefrontal cortex (PFC), whereas auditory verbal hallucinations can be alleviated by cathodal stimulation of Broca’s area (Brunelin et al. 2012; but see Schwippel et al. 2017). In the latter case, cathodal stimulation is intended to reduce hyperactivity in speech-related areas of schizophrenic patients. Similar clinical applications for cathodal tDCS have been used to target excessive activity in clinical conditions such as migraine (Antal et al. 2011; Viganò et al. 2013) or treatment-resistant obsessive–compulsive disorder (Bation et al. 2016).

These promising results notwithstanding, conventional tDCS lacks focal specificity due to large electrode pads and partially unclear fundamental mechanisms. Thus, high-definition (HD)-tDCS has been proposed to increase the low spatial focality of conventional tDCS (Datta et al. 2009; Kuo et al. 2013). This meta-analysis systematically investigates the potential efficacy of reducing cognitive activity with cathodal HD-tDCS across various cognitive domains. We introduce the fundamental mechanisms, extract behavioral results from an extensive literature search on findings from cathodal HD-tDCS and include possible moderators in a multivariate meta-analysis.

## Fundamental mechanism of tDCS in cognition

Conventional tDCS is a form of non-invasive brain stimulation that can alter neural activity and has been observed to produce changes in physiological and behavioral measures (Nitsche and Paulus 2000; Polanía et al. 2018; Priori et al. 1998). If applied over the motor cortex, anodal and cathodal tDCS result in polarity-specific excitability changes in motor-evoked potentials (MEP), with anodal and cathodal stimulation resulting in slightly enhanced and reduced excitability, respectively (Nitsche and Paulus 2000; Polanía et al. 2018; Priori et al. 1998). TDCS has been increasingly used in basic research and clinical investigations, as it offers a way of exploring functionality and causality between variables with low cost and comparably simple application. Thereby, tDCS has been argued to provide further insights into the neurophysiological foundations of cognition and behavior (Filmer et al. 2014). Modulation of performance has also been observed across a multitude of cognitive functions involving attention, learning, and working memory (Coffman et al. 2014; Kuo and Nitsche 2012; Schroeder and Plewnia 2020). Furthermore, due to its potential of inducing lasting changes in brain plasticity when administered repeatedly (Fritsch et al. 2010; Polanía et al. 2018), the therapeutic usefulness of tDCS is increasingly studied in mental disorders, for example, major depressive disorder or schizophrenia (Baeken et al. 2016).

In tDCS, behavioral changes are conventionally caused by placing an anode and cathode electrode directly over the skull and running a weak, direct electrical current of 0.5–2 mA through the underlying cortex. While transcranial magnetic stimulation (TMS), which is another form of non-invasive brain stimulation, can invoke or inhibit actual action potentials in neurons, tDCS largely increases (anodal) or decreases (cathodal) neural resting potentials by either hypo- or hyperpolarization, respectively, thus altering the excitability of neurons in the stimulation area (Fertonani and Miniussi 2017). Notably, this dichotomization of anodal and cathodal tDCS effects oversimplifies the fact that the orientation of neuron populations and cortical folding relative to the current flow specify neuron polarization (Rahman et al. 2013).

According to the anodal-excitation and cathodal-inhibition (AeCi) framework (Nitsche and Paulus 2000), anodal stimulation results in enhanced excitability, while cathodal stimulation diminishes it. However, the AeCi framework was conceived by measuring motor-evoked potentials (MEPs) and may not necessarily translate into the domain of cognition. While the AeCi principle was supported by studies investigating motor functions using MEPs, behavioral results from studies investigating cognitive functions showed a more heterogeneous effect of tDCS as evidenced by a comprehensive meta-analysis on polarity-specific effects (Jacobson et al. 2012). Cognitive studies reported the enhancing effect of anodal tDCS in a similar frequency as motor studies, but the inhibitory effect of cathodal tDCS was found significantly less often (effect probability of 0.81 vs 0.47; (Jacobson et al. 2012)). Furthermore, the mean effect size of cathodal stimulation was significantly smaller than the mean effect size of anodal stimulation. The authors concluded that cathodal tDCS appeared to produce highly heterogeneous results in cognition studies.

Generally, the heterogeneity in the results of tDCS studies is often attributed to intra-individual variability or to technical parameters of the stimulation (Horvath et al. 2014). In meta-analyses, moderator variables can be used to investigate sources of variability. For instance, in the above-mentioned meta-analysis on polarity-specific effects, Jacobson et al. (Jacobson et al. 2012) classified cognitive functions and reported a lack of inhibitory cathodal effects in the language domain, but a more balanced frequency of cathodal inhibition effects in executive functions, memory, and attention. This was argued to reflect the inefficiency of cathodal tDCS to interfere with a rich and distributed neural network (Jacobson et al. 2012), possibly underscored by compensatory neural activity (Schroeder and Plewnia 2016). Notably, another meta-analysis with a focus on inhibitory control even reported differences between two outcomes from two state-of-the-art behavioral paradigms, which seemed to interact with tDCS to slightly varying degrees (Schroeder et al. 2020). Therefore, differences in the functional activation of a neuronal region by a cognitive task may be considered for a moderator analysis of cathodal HD-tDCS effects.

## Meta-analytic findings

Evidence from other meta-analyses supports the notion of heterogeneity or arrive at even more pessimistic views on the efficacy of (cathodal) tDCS (Filmer et al. 2020; Horvath et al. 2015). In a meta-analysis investigating tDCS effects on the dorsolateral prefrontal cortex, evidence indicated that cathodal tDCS showed significant heterogeneity in accuracy measures of cognitive tasks, but not in reaction time measures (Dedoncker et al. 2016). Despite this, the overall effect size in the accuracy subgroup was found to be non-significant. Interestingly, behavioral measures were influenced by the gender distribution of the study as well as the type of sample (healthy vs patients; (Dedoncker et al. 2016)). Cathodal tDCS, however, was not analyzed within healthy and patient subgroups, as no cathodal stimulation was used in clinical populations included for meta-analysis. Differences in the stimulation effect due to the type of sample have been suggested before (Kuo et al. 2014) as brain states are likely to differ between patient and healthy populations, depending on the investigated brain region and cognitive function. For example, a meta-analytic investigation on effects of anodal tDCS on working memory revealed improvements in reaction time measures of cognitive tasks in healthy samples when stimulated before task onset, while improved accuracy was observed in clinical populations that performed on the task during stimulation (Hill et al. 2016).

Furthermore, cathodal tDCS has also not been found to differ from the effects of anodal tDCS in measures of response inhibition (Schroeder et al. 2020), or to produce behavior-enhancing effects under specific circumstances (Schroeder and Plewnia 2016). Moreover, many quantitative reviews have not even included cathodal tDCS in their investigations or did not specify polarity subgroups (Bell and DeWall 2018; Hill et al. 2016; Horvath et al. 2015; Price et al. 2015).

## Technical parameters

Negative results from tDCS studies may relate to the wrong stimulation site(s), ineffective current density in the target region, or other technical parameters. Importantly, technical parameters can relate to outcomes in non-linear ways. For instance, cathodal tDCS over the primary motor cortex areas increased excitability at the target area at high (2 mA) or low (0.5 mA) intensity, while only a moderate current strength of 1 mA corresponded to a decrease in measured excitability (Batsikadze et al. 2013; Jamil et al. 2017). Anodal stimulation, on the other hand, was not observed to be affected by different stimulation intensities and resulted in equivalent facilitatory effects (Jamil et al. 2017). Moreover, to achieve a preferential neuromodulation of a target area, both technical parameters—such as electrode placement, electrode size, and current density—as well as functional parameters—such as cognitive domain, task variation, and duration—should be considered (Bikson and Rahman 2013). Studies have shown that it is not only the placement of the target electrode that significantly affects stimulation effects, but also the placement of the return electrode, as it affects current flow underneath the target (Bikson et al. 2010; Moliadze et al. 2010; Schroeder et al. 2020).

Another source of potential heterogeneity in cognition modulated by conventional cathodal tDCS might stem from the use of bilateral electrode montages, in which the return electrode is placed over the same brain area as the target electrode, but contralaterally over the other brain hemisphere. The results obtained from bilateral stimulation may be difficult to interpret in terms of individual effects of either anodal or cathodal electrodes, as compensation effects are possible when the respective cognitive function is not represented in one location but emerges from a function-specific network of different areas (Bikson et al. 2010; Moliadze et al. 2010). This challenge is avoided in HD-tDCS, which makes use of multiple evenly spread return electrodes to induce focal neuromodulation.

## High-definition tDCS

One of the most prevalent limitations in conventional tDCS configurations is the low spatial precision due to low focality, usually resulting from relatively large electrode sizes and functionally effective return electrodes (Nitsche et al. 2007). As current density is one of the factors that determines (cathodal) tDCS efficacy (Jamil et al. 2017), increased focality could be achieved by reducing target electrode size, but keeping the density constant. Alternatively, increased focality is attained by increasing the size of the reference electrode while maintaining a constant current strength (Nitsche et al. 2007).

Low focality was further supported by studies that simulated current flow from conventional tDCS through a brain (Datta et al. 2009, 2008). Accordingly, a modified electrode montage of five electrodes was suggested, consisting of one target electrode and four return electrodes, which increased stimulation focality substantially (Datta et al. 2009, 2008). Due to its increased spatial precision, the 4 × 1 configuration was termed high-definition (HD) tDCS. Further work corroborated the initial findings on HD-tDCS and demonstrated that the electric current does not spread substantially outside the stimulation area set by the circular placement of return electrodes (Edwards et al. 2013). Moreover, HD-tDCS had comparable polarity-specific effects and the same potential for inducing neuroplasticity in the motor cortex as conventional tDCS (Kuo et al. 2013).

Although conventional tDCS is still more common in clinical and basic research, HD-tDCS is increasingly applied in the context of cognition. As such, one study (Hogeveen et al. 2016) observed comparable effects of anodal HD-tDCS on response inhibition compared to conventional tDCS. The effects of cathodal high-definition stimulation, however, were found to be similarly inconsistent. Risk-based decision-making, for instance, was diminished from cathodal HD-tDCS over the left prefrontal cortex in one study, whereas, anodal HD-tDCS did not differ from sham stimulation (Guo et al. 2018). The performance in social cognition tasks, however, was not affected by cathodal HD-tDCS (Andrew Kenneth Martin et al. 2017a, b). Despite this, behavioral changes were observed in the investigation of executive functions via an emotional counting Stroop task, revealing a facilitation effect of cathodal HD-tDCS (To et al. 2018). Hence, comparable to conventional tDCS, initial results from HD-tDCS studies seem to suggest analogous heterogeneity despite better spatial precision of the stimulation.

Some first evidence on selective effects of HD-tDCS based on variation of stimulation intensity was provided by a recent interventional study with aphasia patients (Fiori et al. 2019). In this study, aphasia patients improved in verb naming following a five-day training augmented with cathodal HD-tDCS at 2 mA intensity, but not at 1 mA. However, as this study featured a multi-session design, results may not necessarily translate to single-session applications of HD-tDCS at different current intensities, because the magnitude of the stimulation effect has been observed to differ between single-session and multi-session designs (Jauch-Chara et al. 2014; Song et al. 2019).

## The present study

There is initial evidence on the efficacy of HD-tDCS in cognition, but its effects on cognitive functions are highly variable and depend on a multitude of experimental and technical factors. Specifically, the application of cathodal HD-tDCS produced inconclusive results across cognitive domains and different stimulation settings. Therefore, the aim of this meta-analysis was a dedicated evaluation of the effects of cathodal HD-tDCS on measures of cognition, while considering experimental and technical parameters as moderator variables. The analysis was performed on studies using single-session, cathodal (HD-)tDCS which evaluated its effects on behavioral outcome measures associated with cognitive functions, e.g., reaction times and accuracy. We used multi-level modelling to include all available effect sizes from individual studies in a meta-analytic model while also considering the dependency amongst them.

Based on previous research, a small negative or null-effect across all studies in the sample was deemed probable, as well as at least moderate heterogeneity. As in previous quantitative reviews, a multitude of pre-defined moderator variables were included in the analysis to explore possible influences on stimulation effects. In the main analysis, we first focus exclusively on studies that employ a 4 × 1 electrode configuration in cathodal polarity to modulate cognition. The analysis is expected to yield some insight into which factors are the most relevant to cathodal HD-tDCS. In an extended analysis with a larger study sample, we also consider a comparable sample of studies that employ conventional tDCS. Accordingly, the results from this meta-analysis might elucidate new directions for future tDCS research in the cognitive domain and contribute to the implementation of more efficient experiments.

## Main analysis: multi-level meta-analysis of cathodal HD-tDCS

### Methods

This meta-analysis has been conducted in agreement with the guidelines of the Preferred Reporting Items for Systematic Reviews and Meta Analyses (PRISMA) (Moher et al. 2009).

#### Eligibility criteria

Studies were eligible for inclusion if they were peer-reviewed and published in a scientific journal. Only studies in English language were considered. One key requirement involved the use and report of cathodal HD-tDCS in human participants and a dedicated control group or condition. The control condition had to be specified as either sham stimulation, an active control stimulation of different polarity or baseline performance. To limit the scope of the analysis and obtain an extensive set of studies, only electrode configurations with a 4 × 1 placement with a central cathodal (i.e., cathodal HD-tDCS) and only cognitive outcomes were considered eligible.

Studies employing stimulation tools other than 4 × 1 HD-tDCS (e.g., conventional tDCS or other electrode designs, tACS, tRNS, or TMS) were not included. Furthermore, it was required that studies reported experimental designs that produce behavioral data, as well as tasks that are clearly connected to distinct cognitive domains (e.g., the Stroop task is associated to executive functions, while the visual perspective-taking task measures aspects of social cognition). Outcome variables could involve reaction times, accuracy measures and other computed scores, depending on the task used as a correlate for the cognitive function in question.

#### Search strategy

The literature search was conducted in the PubMed, Web of Science and Scopus databases. We considered studies with publication dates from 01 January 2000 to 01 June 2021. In all databases, we used the following search terms to extract published articles: “(tDCS OR transcranial direct current stimulation OR HD-tDCS) AND cathodal stimulation AND (cognition OR cognitive function OR performance OR behavior)”. In case additional studies were found by investigating additional sources, these were added manually. To find all studies employing cathodal HD-tDCS in a 4 × 1 stimulation design, we used additional keywords in the Rayyan app (Ouzzani et al. 2016) on the group of studies that were labeled for inclusion after the title-and-abstract screening. The following keywords were used: “High Definition”, “High-Definition”, “HD-tDCS”, “focal” and “4 × 1”.

#### Study selection

We used the web-based Rayyan app for systematic reviews (Ouzzani et al. 2016) to conduct the screening of our search results. After removing duplicates, all remaining studies were filtered in terms of relevance for the present analysis by screening the title and abstract. The title-and-abstract screening was conducted in parallel by two of the authors. Conflicts related to inclusion or exclusion of studies were resolved by subsequent discussion.

**Fig. 1:**
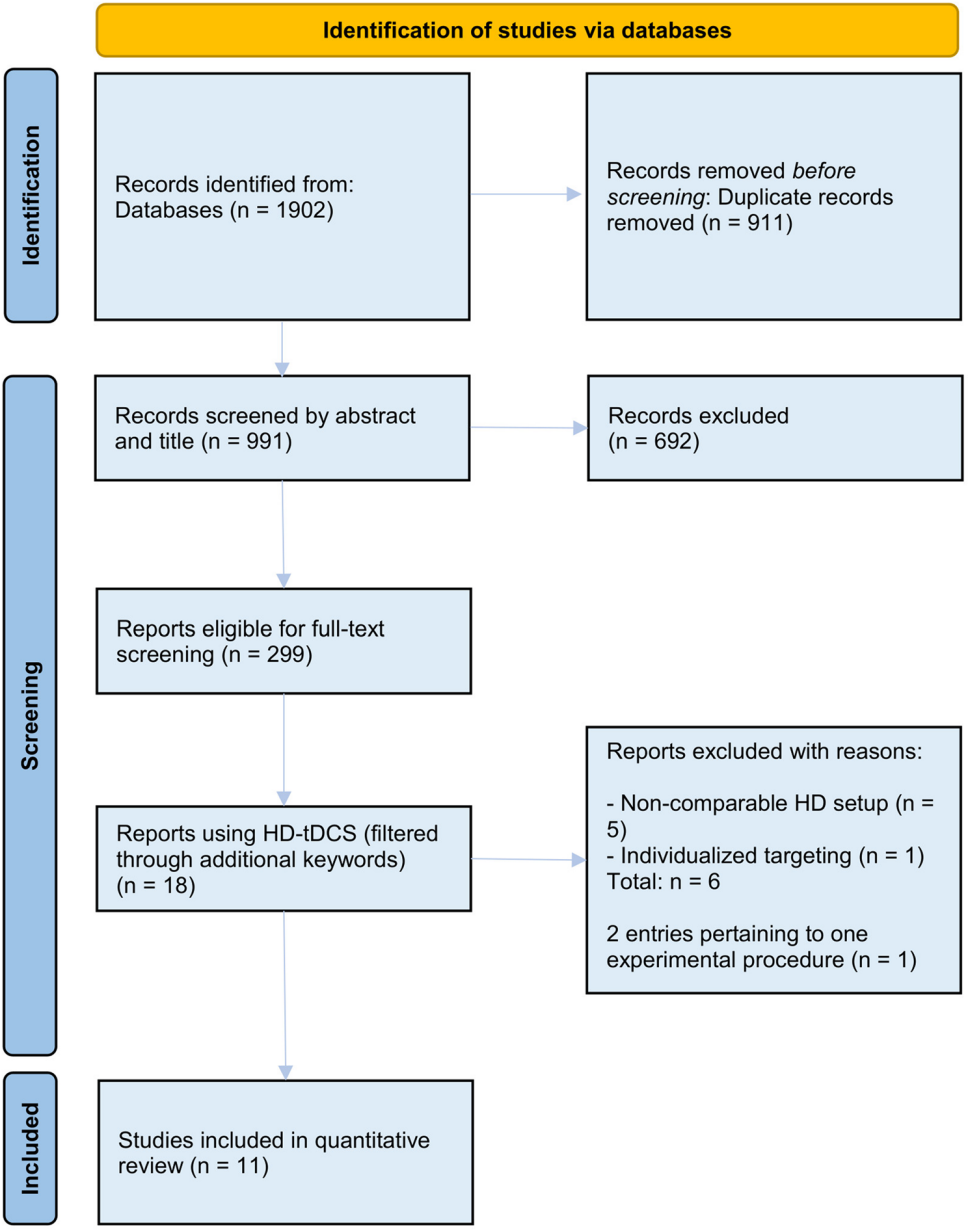
PRISMA flow chart of the literature search

Studies were excluded in this phase, if title and abstract screening revealed that either no cathodal tDCS was administered, or no behavioral outcome variables were measured. Thus, studies reporting only effects of tDCS on physiological measures, e.g. event-related potentials, BOLD-activity or motor-evoked potentials, were excluded. As this meta-analysis focused on the effect of cathodal HD-tDCS on cognitive functions, we also excluded publications whose research questions did not involve cognitive mechanisms, i.e. studies focusing on the motor domain as well as the perceptual domain. We did not limit our sample to studies investigating specific cognitive functions or using specific tasks in their experimental designs, as the aim was to focus on the effect of cathodal HD-tDCS on cognition across domains and tasks. Studies employing a multi-session design of multiple stimulation sessions per participant with the same parameters and conditions were excluded, as the present investigation focuses exclusively on single-session effects of cathodal HD-tDCS. Exceptions were made in those cases where distinct behavioral data from the first stimulation session was available. An overview of the selection process is depicted in Fig. 1.

#### Data extraction

All reported effect sizes were extracted from individual studies and their supplementary materials, and we coded all possible combinations of conditions, tasks, and measures. Comprehensive data were extracted from the final study sample to acquire information from every study according to the moderator variables selected a-priori. For a detailed overview of moderator variables, see Tables 1 and 2.

- Study parameters: experimental design, level of blinding, control condition, cognitive domain
- Sample parameters: sample size, experimental and control group size, percentage of females in the sample, mean age, population (healthy vs. patient)
- Stimulation parameters: target brain region, distance of return electrodes to target electrode, timing (online vs. offline), stimulation intensity, density, density charge, electrode size, stimulation duration, ramp-up time
- Outcome measures: means, standard deviations and/or standard errors of behavioral measures of cognition during active HD-tDCS vs. control condition

**Table 1:** Overview of predefined moderator variables, frequency of effect sizes (*n*) and pooling of levels

Variable	Level	*n*	Pooled level
Level of blinding	Single	11	
	Double	48	
	Not reported	18	
Experimental design	Between-subjects	17	
	Between-subjects with baseline	19	
	Within-subjects	22	
	Mixed	19	
Cognitive domain	Risk-based decision-making	3	a)
	Executive functions	8	a)
	Inhibition	8	a) Executive functions
	Impulsivity	14	
	Language	6	
	Reward learning	6	
	Social cognition	13	b)
	Empathy	4	b)
	Theory of mind	15	b) Social cognition
Target electrode placement	F3	22	
	F4	7	a)
	F6	2	a)
	FC6	4	a) Right prefrontal
	FP2	3	b)
	FZ	20	b) Central prefrontal
	P6	15	
	T7*	4	
Timing	Online	33	
	Offline	44	
Distance between target and return electrode	One*	3	
	Two	18	
	Mixed	19	
	Other	22	
	Not reported	15	
Measure	Reaction time	38	
	Accuracy	18	
	Other	21	
Pathology**	Aphasia	2	
	None	75	
Stimulation intensity (mA)	1	30	
	1.5	7	
	2	40	
Stimulation duration (min)**	13	4	
	20	73	

Subgroups marked with ^*^were excluded from subgroup analyses because of group size (*k* < 5), as they could not be reasonably pooled together with other subgroups

^**^The pre-defined moderator variables “pathology” and “stimulation duration” were excluded from subgroup analyses, as not enough data points were found for subgroups

**Table 2:** List of predefined continuous moderator variables and measuring unit

Predefined moderator	Unit
Electrode size	cm^2^
Current density	A/m^2^
Density charge	kC/m^2^
Age	Mean age of participant sample
Gender	Percentage of females

#### Statistical analysis

The Standardized Mean Difference (SMD; Hedge’s g) was calculated to measure the effect size of cathodal HD-tDCS as compared to controls (sham, baseline performance). Since a multi-level meta-analysis was performed, effect sizes were calculated for every experimental condition with active cathodal stimulation. Thus, multiple effect sizes were assessed per study if its experimental design featured more than one outcome variable, experimental condition, or task. In most cases, experimental designs involved multiple cognitive tasks and also multiple outcome measures per task. These outcome measures were classified as either reaction times (RTs), accuracy or error rate values (ACC), or other task-dependent scores (other). Due to the fact that lower values in one outcome measure represents worse performance, and better performance in another, we adjusted the calculated SMD, so that negative values universally translated to a decrease in performance as an effect of cathodal HD-tDCS, and positive values corresponded to an increase in performance.

If the required statistics for effect size calculation were not reported in the publication, the authors were contacted. If the authors could not provide the data or did not respond, WebPlotDigitizer^1^ was used to extract means and variances from the corresponding figures wherever possible.

**Fig. 2:**
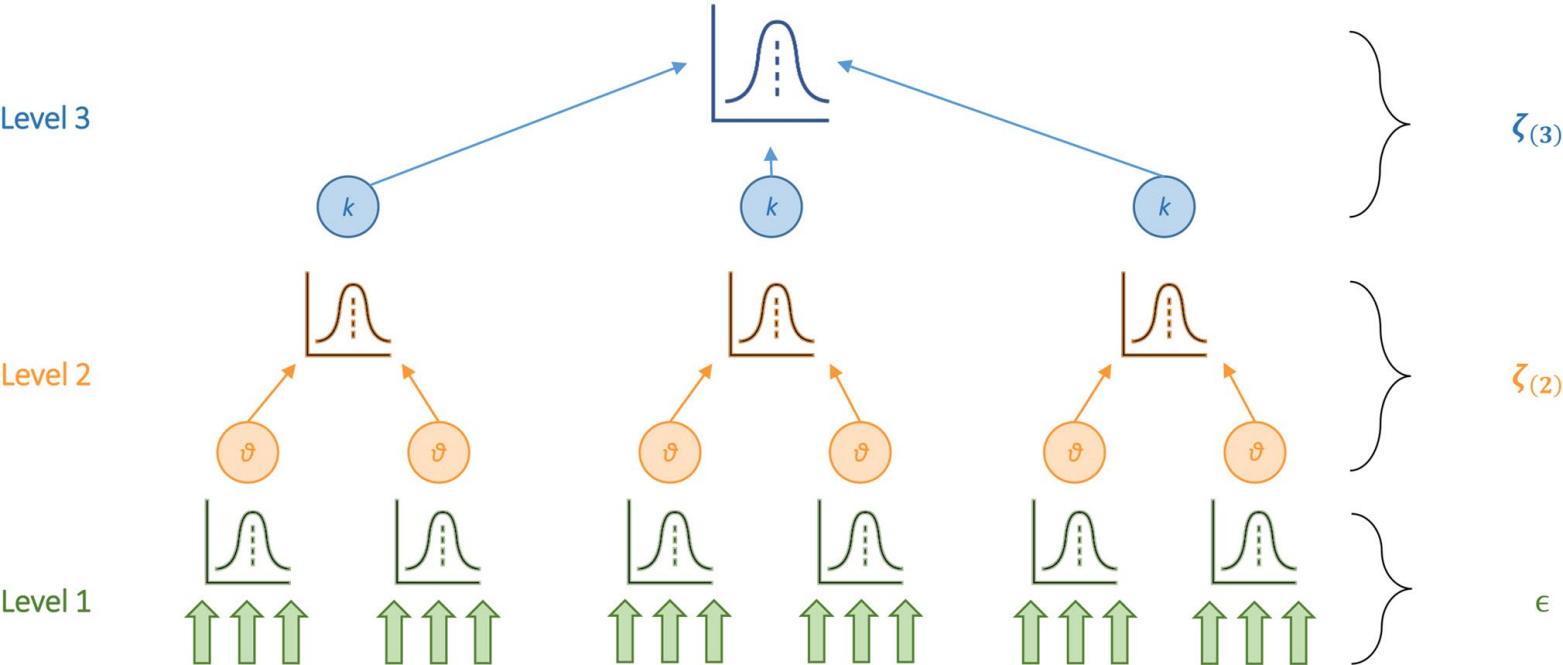
Visual representation of a three-level random-effects model, as used in this study. Results of individual participants are aggregated (Level 1), nested in clusters (Level 2) and pooled to an overall true effect size (Level 3)

The meta-analysis and moderator analysis procedures were performed using the metafor (Viechtbauer 2010) and clubSandwich (Pustejovsky and Tipton 2018) packages in R (v. 4.1.0). A three-level random-effects model was fitted to the data to investigate the overall effect of cathodal HD-tDCS on measures of cognition. The application of random-effects models is based on the assumption that studies included in the sample do not stem from the same population of studies (Riley et al. 2011), which is why it is recommended to be used in the context of experimental psychology and health sciences. The *Q* test (Cochran 1954; Huedo-Medina et al. 2006) was used to assess whether heterogeneity is present in the data, summing the weighted squared deviations of the individual effect sizes from the pooled effect size. To further assess the distribution and magnitude of heterogeneity, a multi-level version of the *I*^*2*^-statistic was used (Cheung 2014). This multi-level *I*^*2*^ approach describes the proportion of variation that is assigned to level 2 or level 3 according to the estimated heterogeneity and within-study variance estimates. Splitting the *I*^*2*^- stastic was accomplished using the *var_comp()* function of the *dmetar* package for R (Harrer et al. 2019). Further, the multivariate three-level model accounts for the non-independence of individual effect sizes within studies, and allows for the separate estimation of within-study (level 2) and between-study (level 3) variance components (Assink and Wibbelink 2016; Cheung 2014; Harrer et al. 2021). As most studies report more than one measure of cognitive function per task, the introduction of level 2 to the model should account for effects that are usually concealed by focusing only on one summary measure per study and between-study heterogeneity. Mathematically (Eq. 1), the model can be expressed as

1 $$\hat{\theta }_{{{\text{ij}}}} = \mu + \zeta_{{\left( 2 \right){\text{ij}}}} + \zeta_{{\left( 3 \right){\text{ij}}}} + \in_{{{\text{ij}}}} ,$$

where $\hat{\theta }_{{{\text{ij}}}}$ is an estimate of the true effect size *i* nested in study *j*, with $\zeta_{{\left( 2 \right){\text{ij}}}}$ and $\zeta_{{\left( 3 \right){\text{ij}}}}$ describing the within-study heterogeneity on level 2 and the between-study heterogeneity on level 3, respectively (see Fig. 2 for a visual representation). The mean of the distribution of true effect sizes is represented by *µ*, while $\in_{{{\text{ij}}}}$ represents the sampling error.

The assumption of non-independence of effect sizes within a cluster implies a correlation of effect sizes. This is relevant not only in studies using within-subjects designs, but also all studies using several different tasks that measure the same cognitive function. In an ideal case, the correlation of effect sizes within clusters can be calculated through a variance–covariance matrix based on the raw data. However, as this kind of data is only seldom available, we estimated the variance–covariance matrix by assuming a correlation of the sampling errors of the effect sizes to be *ρ* = 0.60. Based on that, cluster robust variance estimations of standard errors and hypothesis tests were calculated to account for within-study dependence (Pustejovsky and Tipton 2021). Wald-type tests were used to assess the effect of pre-defined moderator variables (Pustejovsky and Tipton 2021). The reported results of the main meta-analysis as well as the moderator analyses are based on the cluster-robust variance estimation as outlined above.

The effect of moderator variables on cathodal HD-tDCS outcome was investigated by individual submission of the moderators to the three-level random-effects model (see Tables 1 and 2 for a list of individually submitted moderator variables). Moderators whose contribution to the model was significant were subsequently submitted to a full three-level model simultaneously. Subgroups of discrete moderator variables were only considered if the number of effect sizes per subgroup was *k* ≥ 5 (Van Houwelingen et al. 2002). Although subgroup effect sizes were pooled if *k* < 5, the subgroup “one” of the return placement variable for HD-tDCS as well as the subgroup “T7” of the target region variable could not be pooled reasonably, and were therefore excluded from subgroup analysis.

Finally, small-study effects (possibly indicating publication bias) were assessed using Egger’s regression test (Egger et al. 1997; Sterne and Egger 2006) by submitting the sampling variance of the effect sizes to the random-effects model as a moderator. A significant deviation of the intercept from zero would indicate an asymmetry in the relationship between the precision and size of the studies, and therefore indicate bias. Further, outliers in the data were investigated by analyzing the hat values (i.e. diagonal elements of the hat matrix) and standardized residual values. A data point was considered an outlier if its hat value was greater than two times the average hat value (i.e. influential), and standardized residual values were greater than 3 (Aguinis et al. 2013; Viechtbauer and Cheung 2010).

## Results

**Fig. 3:**
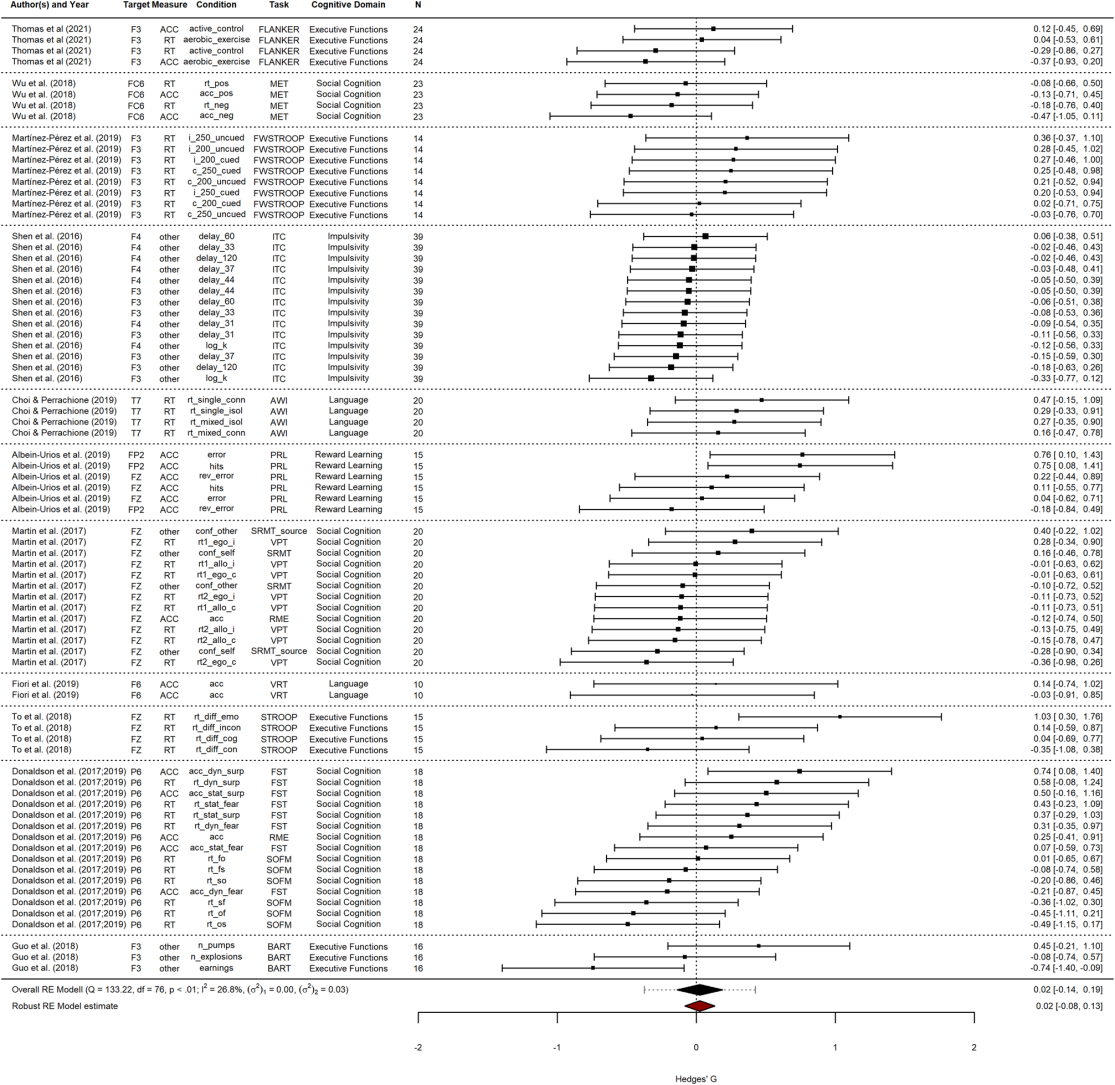
Summary of effect sizes for single-session cathodal HD-tDCS on behavioral measures of cognition. Multiple dependent effect sizes from single studies (e.g., different task conditions, outcome variables) were modeled on Level 1 and possible moderator variables (e.g., target, measure, task, domain) were submitted to multi-level meta-analysis. *N* sample size

After the screening process, 11 studies with a 4 × 1 HD-tDCS configuration and cathodal polarity remained in the sample for analysis. This resulted in a total of 77 individual effect sizes. In sum, 535 participants were recruited across all studies, ranging from 20 to 115 participants per publication (median = 45). Experimental and control group sizes for included experiments ranged from 10 to 39 participants (median = 20), including both between- and within-subjects designs. Most effects were related to social cognition (*n* = 32), followed by executive functions (*n* = 19), impulsivity (*n* = 14), language, and reward learning (each *n* = 6). Regarding technical parameters, electrode sizes ranged from 1.14 to 5 cm^2^ and stimulation intensities between 1 and 2 mA were employed; Table 3 presents an overview of the included studies. Figure 3 shows the individual effect sizes, as well as the pooled overall effect size.

**Table 3:** Study sample information, number of effect sizes (*k*) and total number of participants (i.e., across experiments / conditions)

Author (Year)	*k*	Cognitive domain	Target	Timing	Design	*N*
Thomas et al. (2021)	4	Executive functions	F3	Offline	Within-subjects	115
Wu et al. (2018)	4	Social cognition	FC6	Offline	Within-subjects	24
Martínez-Pérez et al. (2019)	8	Executive functions	F3	Online	Between	29
Shen et al. (2016)	14	Impulsivity	F3, F4	Online	Within-subjects	39
Choi & Perrachione (2019)	4	Language	T7	Online	Mixed	60
Albein-Urios et al. (2019)	6	Reward learning	FP2, FZ	Offline	Between-subjects	52
Martin et al. (2017a, b)	13	Social cognition	FZ	Online	Mixed	40
Fiori et al. (2019)	2	Language	F6	Online	Mixed	20
To et al. (2018)	4	Executive functions	FZ	Offline	Between-subjects*	45
Donaldson et al. (2019)	15	Social cognition	P6	Offline	Between-subjects*	53
Guo et al. (2018)	3	Executive functions	F3	Online	Between-subjects	58

Target is indicated in reference to the international 10–20 system of electrode placement

^*^Between-subjects design with baseline comparison

### Pooled effect size and heterogeneity

Fitting the multi-level random-effects model to all 77 effect sizes revealed an overall non-significant effect of cathodal HD-tDCS on measures of cognition of *g* = 0.024 [SE = 0.055, CI (95%) = (− 0.103; 0.150), *t* = 0.427, *p* = 0.680]. The *Q*test for true heterogeneity was highly significant, *Q*_E_(76) = 133.23, *p* < 0.0001, with estimated variance components of *σ*^2^_Level__3_ < 0.001 and *σ*^2^_Level__2_ = 0.056. Thus, *I*^*2*^_Level__3_ < 0.1% of the total variation could be attributed to between-study heterogeneity, and *I*^2^_Level__2_ = 26.8% to within-study heterogeneity.

### Moderator analysis

The influence of pre-defined moderator variables on the effect of cathodal HD-tDCS was investigated by systematically submitting them to the random-effects model individually, resulting in one model per moderator. Results for residual heterogeneity and moderator tests for discrete and continuous moderator variables are shown in Table 4. In the present sample of studies, no moderator showed a significant influence on stimulation effects, whereas all tests for heterogeneity were highly significant, indicating that heterogeneity was still present after moderator submission. Thus, no moderator could be included in a full model simultaneously.

**Table 4:** Tests for heterogeneity and moderator tests for individual moderator submissions to the multi-level random-effects model

Moderator	*σ*^2^_Level 2_	*Q*_E_	df	*p*_Q_	*F*	df	*p*_F_
Experimental design	0.034	130.7446	73	< .0001***	2.97	3	0.279
Blinding	0.034	132.7431	74	< .0001***	0.32	2	0.753
Cognitive domain	0.050	106.5317	54	< .0001***	1.37	2	0.418
Target region	0.035	125.3812	69	< .0001***	0.41	3	0.775
Timing	0.034	133.2222	75	< .0001***	0.01	1	0.933
Electrode distance	0.023	113.8478	70	.0007***	0.06	3	0.972
Measure	0.035	132.0243	74	< .0001***	0.17	2	0.853
Stimulation intensity	0.034	131.6208	74	< .0001***	3.06	2	0.22
Electrode size	0.034	132.1122	75	< .0001***	4.32	1	0.101
Current density	0.034	132.2976	75	< .0001***	3.95	1	0.13
Density charge	0.034	132.7747	75	< .0001***	1.91	1	0.250
Age	0.035	132.569	73	< .0001***	0.92	1	0.375
Gender	0.034	132.7958	75	< .0001***	0.56	1	0.493

Test statistic, degrees of freedom and respective *p* values are provided

Results for cognitive domain are based on the model fit after exclusion of factor levels

^*^p < .05

^**^p < .01

^***^p < .001

For the moderator cognitive domain, two of the pooled domains were exclusively related to one individual study each. As the between- and within-studies variance components could not be resolved in those cases, those levels were dropped from the moderator analysis. No significant moderation effect was observed, *F*(1) = 0.376, *p* = 0.275. Notably, the corresponding pooled effect sizes of the excluded moderator subgroups impulsivity (*g* =  − 0.09) and reward learning (*g* = 0.28) were among the most excessive mean effect sizes in the current sample (see Table 5 for detailed information in individual subgroup effect sizes). For the moderator stimulation duration, only *k* = 4 effect sizes from a single study were obtained that deviated from a stimulation duration of 20 min; accordingly, no moderator analysis was performed. Similarly, only *k* = 2 effect sizes were obtained that were based on a patient sample, while the rest of the data corresponded to healthy participants. Thus, no moderator analysis was performed on the pathology moderator variable.

**Table 5:** Detailed overview of pooled effect sizes on subgroup level of discrete moderator variables

Moderator subgroups	*k*	Estimate	SE	*t*	*p*
Experimental design					
Between-subjects	17	0.124	0.125	0.996	.425
Between-subjects baseline	19	0.147	0.058	2.531	.240
Within-subjects	22	− 0.129	0.038	− 3.411	.088
Mixed	19	0.102	0.129	0.796	.522
Blinding					
Single	11	− 0.069	0.123	− 0.561	.633
Double	48	0.065	0.068	0.951	.389
Not reported	18	0.029	0.177	0.165	.896
Cognitive Domain					
Executive functions	19	0.017	0.089	0.191	.861
Impulsivity	14	− 0.088	−	−	−
Language	6	0.221	0.112	1.971	.299
Reward Learning	6	0.284	−	−	−
Social Cognition	32	− 0.057	0.082	− 0.695	.559
Target brain area					
Central prefrontal	20	0.107	0.102	1.045	.409
Right prefrontal	13	− 0.035	0.066	− 0.523	.652
F3	22	− 0.099	0.065	− 1.521	.225
P6	15	0.076	0.271	0.281	.826
Timing					
Online	44	0.019	0.073	0.263	.806
Offline	33	0.030	0.097	0.305	.776
Electrode distance					
Two	18	0.030	0.177	0.172	.892
Mixed	19	− 0.030	0.100	− 0.300	.815
Other	22	0.073	0.125	0.582	.602
Nr	15	− 0.017	0.043	− 0.391	.763
Measure					
Reaction time	38	− 0.001	0.071	− 0.014	.989
Accuracy	18	0.082	0.123	0.667	.531
other	21	0.010	0.094	0.108	.924
Stimulation intensity					
1 mA	30	0.024	0.073	0.329	.761
1.5 mA	7	− 0.177	0.044	− 3.997	.156
2 mA	40	0.095	0.107	0.894	.436

Number of effect sizes per subgroup (*k*), Hedge’s g (SMD), standard error, test statistic and corresponding *p* value are shown

### Sensitivity analyses

**Fig. 4:**
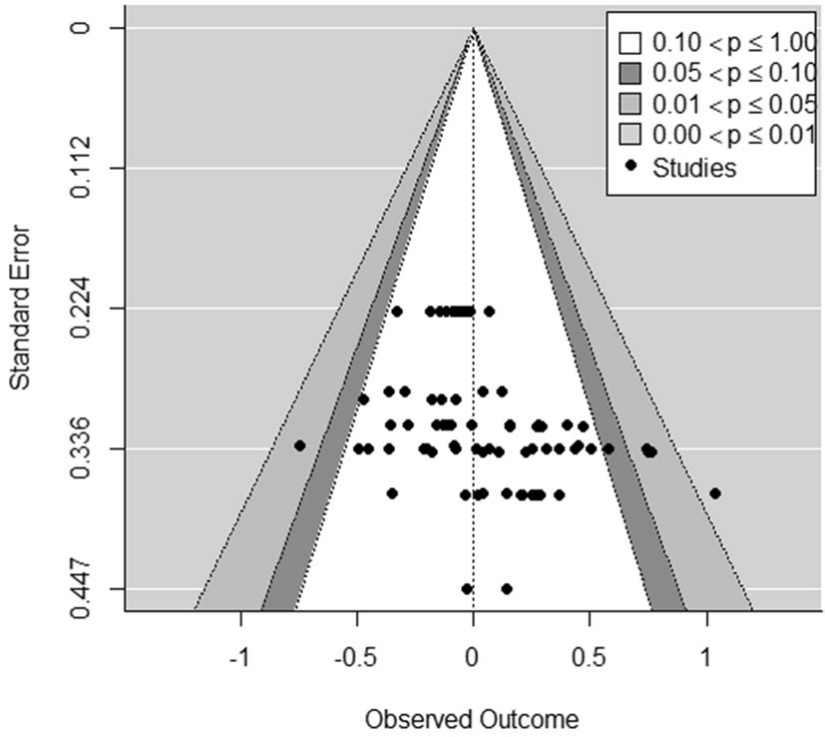
Funnel plot of effect sizes

We used an approximation of Egger’s regression test (Egger et al. 1997; Sterne and Egger 2006) to assess publication bias by submitting the sampling variance of the effect sizes as a moderator variable to the multi-level random-effects model (see Fig. 4 for a funnel plot). The results indicate the presence of publication bias in our data at *p* < 0.05. We did not detect any influential outliers in our data set.

## Extended analysis: cathodal conventional vs. HD-tDCS

### Methods

To frame the findings of our focused multi-level analysis on cathodal HD-tDCS in a more general framework with a larger number of studies, we included a comparable set of studies using conventional cathodal tDCS in an extended analysis. The same eligibility criteria and search terms were employed but keywords for HD-tDCS were omitted. Out of 281 eligible studies, a researcher first matched studies based on the investigated cognitive domain to the set of HD-tDCS studies (reported in the main analysis) and then randomly selected a respective number of studies, yielding a comparable set of 11 studies with cathodal tDCS.

#### Statistical analysis

Effect sizes from all studies were submitted to statistical analysis analogue to Study 1. In addition, we report results from the additional moderator “Stimulation Setup” (HD-tDCS vs. conventional tDCS). An overview of all moderators is provided in Supplementary Table 1.

## Results

**Fig. 5:**
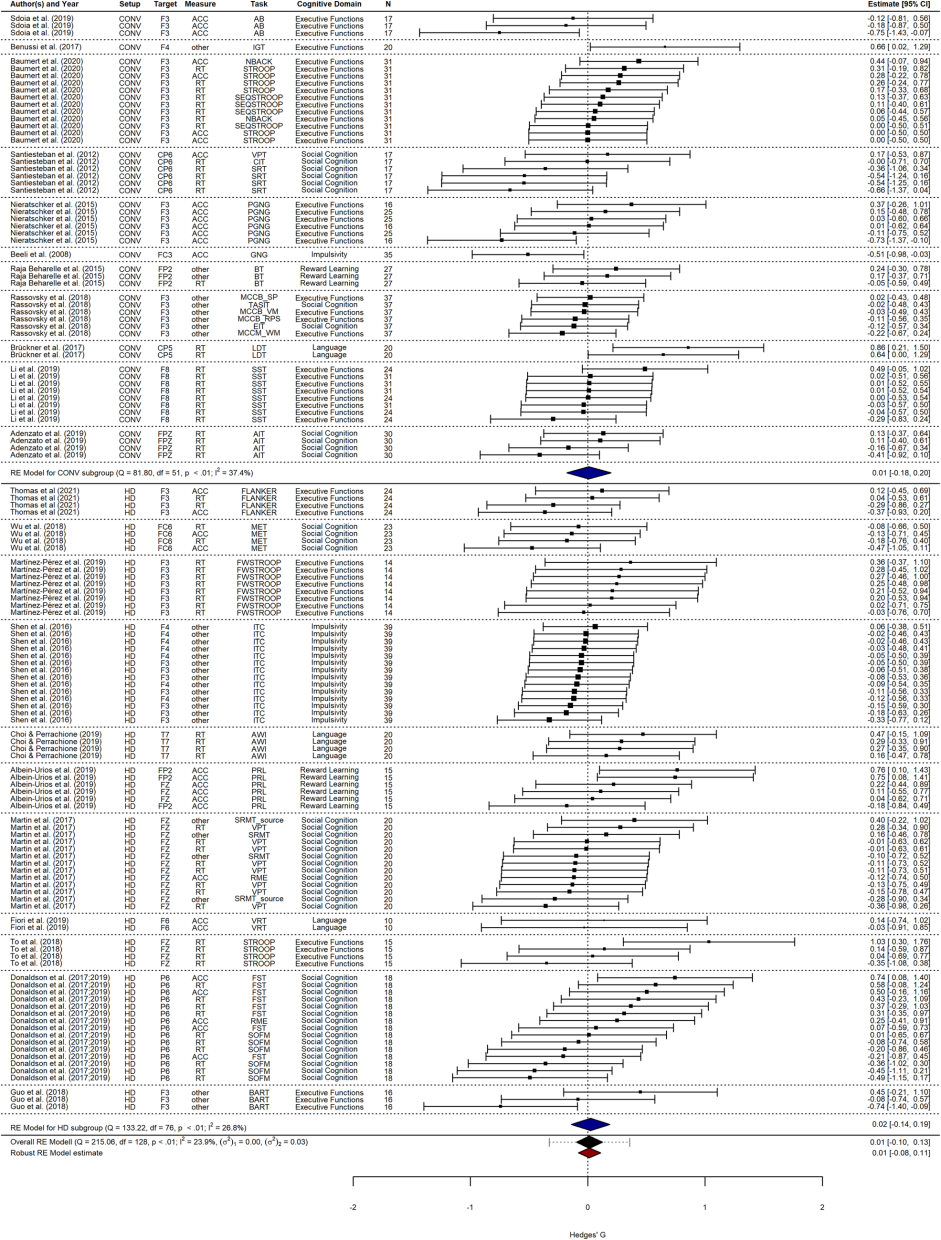
Summary of effect sizes for single-session conventional cathodal tDCS or cathodal HD-tDCS on behavioral measures of cognition. Results are grouped and summarized by setup. Multiple dependent effect sizes from single studies (e.g., different task conditions, outcome variables) were modeled on Level 1 and possible moderator variables (e.g., target, measure, task, domain) were submitted to multi-level meta-analysis. *N* sample size

We included 11 studies with a conventional tDCS configuration and 11 studies with a 4 × 1 HD-tDCS configuration and cathodal polarity for analysis. This sample contained 129 reported effect sizes. Sample sizes were slightly larger for conventional tDCS (617 participants, median = 59; overall: 1152 participants, median = 52). Experimental and control group sizes for included experiments ranged from 10 to 39 participants and were larger for conventional tDCS (median = 30) than for HD-tDCS (median = 20). Studies were recruited to match domains from the HD sample. In this analysis, effect sizes were mostly related to executive functions (*n* = 53), followed by social cognition (*n* = 44), impulsivity (*n* = 15), reward learning (*n* = 9), and language (*n* = 8). Regarding technical parameters in the conventional tDCS configurations, electrode sizes ranged from 7.07 to 35 cm^2^ and stimulation intensities between 1–2 mA were employed; Supplementary Table 2 presents an overview of the included studies. Figure 5 shows the individual effect sizes, as well as the pooled overall effect size.

### Pooled effect size and heterogeneity

Fitting the multi-level random-effects model to all 129 effect sizes from HD and conventional cathodal tDCS revealed an overall non-significant effect of cathodal (HD-)tDCS on measures of cognition of *g* = 0.014 [SE = 0.049, CI (95%) = (− 0.087; 0.116), *t* = 0.285, *p* = 0.779]. The Q-test for true heterogeneity was highly significant, *Q*_E_(128) = 215.85, *p* < 0.0001, with estimated variance components of *σ*^2^_Level__3_ < 0.001 and *σ*^2^_Level__2_ = 0.027. Thus, *I*^*2*^_Level__3_ < 0.1% of the total variation could be attributed to between-study heterogeneity, and I^2^_Level__2_ = 24.1% to within-study heterogeneity.

### Moderator analysis

The influence of moderator variables on the effect of cathodal (HD-)tDCS was investigated by systematically submitting them to the random-effects model individually, resulting in one model per moderator.

Results for residual heterogeneity and moderator tests for discrete and continuous moderator variables are shown in Supplementary Table 3, pooled effect size estimates are provided in Supplementary Table 4. Among the investigated moderators, the extended analysis showed a significant moderation of cathodal (HD-) tDCS effects by tDCS intensity, *F*(2, 9.78) = 4.96, *p* = 0.033. This moderated effect was driven by a negative mean effect size for an intensity of 1.5 mA (*g* = − 0.237), but not for 1 mA (*g* = 0.077) or 2 mA (*g* = 0.086).

In a full model including the significant moderator “intensity”, heterogeneity was reduced only slightly *Q*_E_(126) = 208.25, *p* < 0.0001, with estimated variance components of *σ*^2^_Level__3_ < 0.001 and *σ*^2^_Level 2_ = 0.023. No further moderator showed a significant influence on stimulation effects, whereas all tests for heterogeneity were highly significant, indicating that heterogeneity was still present after moderator submission.

Noteworthy, the entire sample comprised sufficient studies for the moderator analysis on cognitive domain to include the levels impulsivity (*g* = − 0.21) and reward learning (*g* = 0.19). Nevertheless, the moderator test was statistically not significant, *F*(5) = 1.653, *p* = 0.151. Finally, the test for the moderator “Stimulation Setup” was statistically not significant, *F*(1) = 0.031, *p* = 0.863, and mean cathodal tDCS effect sizes were comparable for HD and conventional tDCS (Fig. 5). See Supplementary Table 4 for detailed information in individual subgroup effect sizes.

### Sensitivity analyses

There were no influential outliers in the extended data set. Furthermore, results did not indicate funnel asymmetry (Egger’s test: *p* = 0.22). A funnel plot is shown in Supplementary Fig. 1.

## Discussion

HD-tDCS is a relatively focal, novel non-invasive brain stimulation method with the potential to investigate causal contribution of specific cortical brain regions to cognition, language, and behavior. With cathodal HD-tDCS, neural excitability is assumed to be reduced (Kuo et al. 2013; but see Pellegrini et al. 2020). Behaviorally, this can lead to a variety of outcomes such as increased impulsivity (Shen et al. 2016), reduced inhibitory tagging (Martínez-Pérez et al. 2019), or diminished risky decision-making (Guo et al. 2018). The present multi-level meta-analysis systematically assessed studies that employed cathodal HD-tDCS to modulate cognition and language as evidenced by behavioral outcomes. Descriptively, studies employed a large variety of standard cognitive tasks (e.g., Stroop task, Flanker task, reading the mind in the eyes test) but also more specific paradigms such as an affective mentalizing and face processing task. Studies reported between 2 and 15 effect sizes and to avoid type-1-errors while also considering variation from nested effect sizes within study, we used three-level modeling of variability at within-study and between-study levels. Despite the relatively fixed 4 × 1 electrode setup, electrode sizes ranged from 1.14 to 5 cm^2^ and stimulation intensities between 1 and 2 mA were employed. The meta-analytic results confirmed the variability inherent to cathodal HD-tDCS: There was no significant overall behavioral effect (Hedges *g* = 0.02), but a large variability in behavioral response to cathodal HD-tDCS. At large, these findings are in line with the variability observed in conventional tDCS (Jacobson et al. 2012).

Most intriguingly, the multi-level meta-analysis enabled a differentiation of between-study and within-study heterogeneity in the present analysis. Since most HD-tDCS studies investigated several behavioral indices or even distinct tasks, a multitude of dependent effect sizes under different conditions could be extracted from the studies. Interestingly, the variance in the sample can be attributed to within-study heterogeneity and to between-study heterogeneity by multi-level meta-analysis (Pustejovsky and Tipton 2021). In this regard, 77 effect sizes from 11 studies were almost exclusively attributed to within-studies heterogeneity, whereas almost no between-study heterogeneity was observed here (< 1%). This finding exceeds the usually smaller between-study variance as reported in a previous systematic review across multi-level meta-analytic models in behavioral and social sciences (Fernández-Castilla et al. 2020). Of course, the present result needs consideration and recapitulation in the future when higher samples of cathodal HD tDCS studies are available. Of note, it should be considered that moderator variables such as outcome measure did not substantially increase the proportion of between-study variance in this meta-analysis. If this pattern of level 2 and level 3 heterogeneity holds also in future multi-level meta-analytic models of tDCS effects, it may indicate a high intra-individual variability in response to tDCS that seems to depend on specific study conditions.

The present results also reverberate with the findings from the previous meta-analysis on conventional tDCS (Jacobson et al. 2012) with regards to an overall null effect of cathodal tDCS on cognition, but a large residual heterogeneity. Although the results at first seem inconsistent with the AeCi model of tDCS, we wish to highlight that this model should be understood in terms of direct physiological tDCS effects, but not in terms of its behavioral consequences. Conventional tDCS studies have shown that reduced brain activity from cathodal tDCS can result in performance improvements (Schroeder and Plewnia 2016). The same pattern seems to hold for HD-tDCS as well, at least with regard to the limited number of available studies that were available for the purpose of this study.

Among the possible moderators of the cathodal HD-tDCS response, we could not substantiate significant moderators in our analysis. However, descriptively, heterogeneity seemed reduced when considering cognitive domain, electrode size, electrode distance, target region, stimulation intensity, and current density. Further exploration of the coefficients suggested a possible negative influence with larger negative (inhibitory) effect sizes for the largest target electrodes (here: 4–5 cm^2^). Conversely, a positive coefficient for current density suggested larger positive effect sizes with higher current density. Furthermore, descriptively, the largest stimulation intensity (2 mA) had a more positive effect size (*g* = 0.095) than the lower intensities (1.5 mA: *g* = − 0.177, 1 mA: *g* = 0.024; see Supplementary Table 1). A plausible physiological explanation for this finding is the non-linearity of cathodal tDCS effects (Batsikadze et al. 2013; Jamil et al. 2017), which should translate to HD-tDCS as well.

We had to prune three moderators in the process of analysis: task duration, pathology, and cognitive domain. Regarding task duration, only one study (Choi and Perrachione 2019) employed a shorter task duration (13 min) and was situated in the language domain. In their study, similar behavioral effects of anodal and cathodal HD-tDCS were interpreted as a result of different neuroplastic mechanisms that would both disrupt excitation-inhibition balance in different ways (Choi and Perrachione 2019). Most fundamental studies on excitability changes from varying durations of cathodal tDCS were conducted with shorter intervals (e.g., 5–18 min) but led to comparable excitability reductions (e.g., Fricke et al. 2011; Monte-Silva et al. 2010). Interestingly, in the extended analysis comprising also effect sizes from conventional cathodal tDCS, the moderator variable task duration could be included, although very little studies contributed to non-significant moderator test. Descriptively, the largest negative effect size for cathodal tDCS emerged for durations longer than 20 min.

Regarding cognitive domain, only single studies investigated effects of cathodal HD-tDCS on impulsivity, yielding a small negative effect (Shen et al. 2016) whereas another single study investigated effects of cathodal HD-tDCS on reward learning showed a moderate positive effect (Albein-Urios et al. 2019). Domain-specific differences have been observed in quantitative reviews before (Dedoncker et al. 2016; Jacobson et al. 2012), and the present results support the idea that the effects of cathodal tDCS are dependent on the investigated cognitive function. Interestingly, cathodal stimulation might also improve language function by means of reducing noise in semantic processing (Brückner and Kammer 2017), which is consistent with the positive effect of cathodal HD-tDCS over Broca’s region observed in one study (Choi and Perrachione 2019). In this regard, first results from Aphasia patients were reported who improved in verb naming only following 2 mA, but not 1 mA cathodal HD-tDCS over Broca’s region (Fiori et al. 2019). Notably, their study comprised repeated stimulation across five days whereas, for comparability, only single-session effects were investigated in our analysis. Nevertheless, more results from patients in systematic single-session and multi-session designs are required to bolster the initial optimistic findings.

Although the lack of other significant moderator effects may seem surprising at first, this should not be interpreted as a lack of the respective parameters’ influences on tDCS response in general. Rather, the variability among the moderators themselves in the present sample, the overall small number of studies, and the specific characteristics of included effect sizes may be considered. For instance, only very few studies investigated cathodal stimulation at an intensity of 1.5 mA; thus, although this intensity was descriptively most likely to induce an inhibition effect, more systematic studies will be required. Accordingly, with a larger sample in the extended analysis, we could corroborate this observed trend. The extended analysis comprising all 22 studies with cathodal conventional or HD-tDCS showed the strongest inhibition effects at the intensity of 1.5 mA. For future research with cathodal HD-tDCS, experimental manipulation of such technical parameters also in cognitive tasks may be highly informative.

It is noteworthy to consider some limitations of this meta-analysis. As noted above, only a relatively small number of studies were available and more research in this domain is required. We addressed this limitation in an extended analysis that also included studies with conventional parameters. However, more data with better comparable stimulation configurations are required. It is clear that the optimal electrode positions for a specific task has to be elaborated in different studies. Furthermore, replication studies are also required throughout the investigated domains. Here, we focused on the 4 × 1 HD-tDCS configuration, but more complex model-based electrode montages were not included to maintain comparability of the intervention. Such montages become increasingly more common in HD-tDCS studies, and can include up to 9 electrodes, whose placement and polarity is based on simulations of current flow for optimal stimulation conditions over the brain region of interest (e.g., Maldonado et al. 2019). Moreover, despite a strict technical inclusion requirement, studies differed with regard to the cognitive constructs that were assessed and even with regard to their operationalization in behavioral tasks. With that in mind, another meta-analytic strategy may consider task parameters more strictly as inclusion criterion; in fact, a previous univariate meta-analysis could differentiate between two closely related measures of inhibitory control (Schroeder et al. 2020). However, since very few studies with cathodal HD-tDCS were available, this synthesis presented the most comprehensive analysis and future research may focus on domain-specific effects.

To conclude, cathodal HD-tDCS effects on cognition appear to be variable and heterogeneous, rendering it an interesting, albeit difficult to interpret neurostimulation technique for behavioral studies. Target electrode size was descriptively related to more pronounced inhibitory effects, but larger sample sizes are needed to systematically expose the interplay of electrode size with current density and HD-tDCS intensity. Future multi-level meta-analyses may be appropriate to model multiple dependent variables in tDCS studies and to investigate between- and within-study variance also with larger samples. Nevertheless, equipped with the unique ability to decrease cortical excitability in high spatial focality, future studies may include cathodal HD-tDCS to identify effective and in-effective stimulation protocols.

## Supplementary Information

Supplementary file1 (TXT 0 kb)

Supplementary file2 (JPEG 94 kb)

## Data Availability

Data and scripts can be retrieved from: https://osf.io/ye8cr/.

## Footnotes

1 See Rohatgi (2020)
