# Supplementary figures and images for: More focal, less heterogeneous? Multi-level meta-analysis of cathodal high-definition transcranial direct current stimulation effects on language and cognition
Source: J Neural Transm (Vienna). 2022 May 18;129(7):861–78. doi: 10.1007/s00702-022-02507-3 (PMC9217872; doi:10.1007/s00702-022-02507-3)

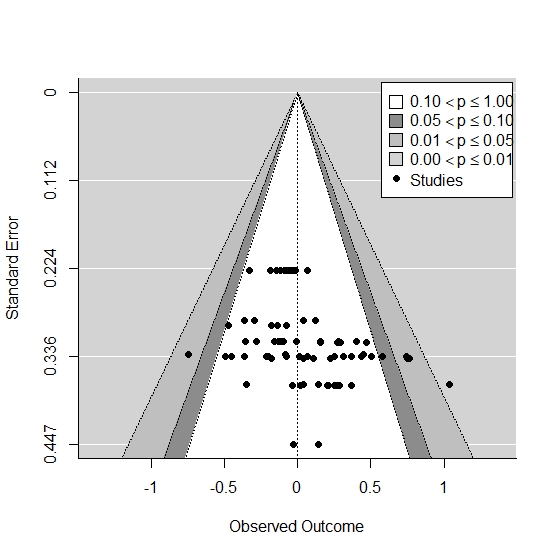

Supplement: Supplementary file 2 — Supplementary file2 (JPEG 94 kb) [file 702_2022_2507_MOESM2_ESM.jpeg]
